# Genetic Diversity and Selection Signatures Within Diannan Small-Ear Pigs Revealed by Next-Generation Sequencing

**DOI:** 10.3389/fgene.2020.00733

**Published:** 2020-07-30

**Authors:** Fen Wu, Hao Sun, Shaoxiong Lu, Xiao Gou, Dawei Yan, Zhong Xu, Zhenyang Zhang, Qamar Raza Qadri, Zhe Zhang, Zhen Wang, Qiang Chen, Mingli Li, Xiaoyi Wang, Xinxing Dong, Qishan Wang, Yuchun Pan

**Affiliations:** ^1^Department of Animal Breeding and Reproduction, College of Animal Sciences, Zhejiang University, Hangzhou, China; ^2^Department of Animal Science, School of Agriculture and Biology, Shanghai Jiao Tong University, Shanghai, China; ^3^College of Animal Science and Technology, Yunnan Agricultural University, Kunming, China

**Keywords:** Diannan small-ear pigs, population structure, genetic diversity, inbreeding coefficients, selection signatures

## Abstract

Genetic characterization of Chinese indigenous pig breeds is essential to promote scientific conservation and sustainable development of pigs. Here, we systematically surveyed the genomes of 75 unrelated Diannan small-ear (DSE) pigs from three diverse regions (Yingjiang County, Jinping County, and Sipsongpanna in Yunnan Province) to describe their population structures, genetic diversity, inbreeding coefficients, and selection signatures. First, these individuals were sequenced and genotyped using the genome reducing and sequencing (GGRS) protocol. A total of 438,038 autosomal single-nucleotide polymorphisms (SNPs) were obtained and used for subsequent statistical analysis. The results showed that these DSE pigs were clearly differentiated into three separate clades revealed by the population structure and principal component analysis, which is consistent with their geographical origins. Diannan small-ear pigs owned lower genetic diversity when compared with some other pig breeds, which demonstrated the need to strengthen the conservation strategies for DSE pigs. In addition, the inbreeding coefficients based on runs of homozygosity (ROH) length (*F*_ROH_) were calculated in each ROH length categories, respectively. And the results indicated that the ancient (up to 50 generations ago) inbreeding had greater impacts than recent (within the last five generations) inbreeding within DSE pigs. Some candidate selection signatures within the DSE pig population were detected through the ROH islands and integrated haplotype homozygosity score (iHS) methods. And genes associated with meat quality (*COL15A1*, *RPL3L*, and *SLC9A3R2*), body size (*PALM2-AKAP2*, *NANS*, *TRAF7*, and *PACSIN1*), adaptability (*CLDN9* and *E4F1*), and appetite (*GRM4*) were identified. These findings can help to understand the genetic characteristics and provide insights into the molecular background of special phenotypes of DSE pigs to promote conservation and sustainability of the breed.

## Introduction

In China, there were more than 80 indigenous pig breeds registered according to the second national census of local pig breeds (2004). Diannan small-ear (DSE) pigs, a specific Chinese native pig breed, are raised in the southern areas of Yunnan province with subtropical climate. The indigenous animals under subtropical climate exhibit heritable adaptations to high temperature and humidity due to natural selection. Therefore, DSE pigs might be the ideal animal models for the research on the diseases related to human. Besides, DSE pigs are well-known for their better meat quality and more fat deposition than Western pigs (Wang et al., 2015). During the long-term breeding process, DSE pigs have been naturally differentiated into mini type and large type (Liu, 2010). And the common types of coat color in DSE pigs include both whole-body black and six-white-point (Lü et al., 2016). Diannan small-ear pigs own the abundant phenotypic traits and superior commercial traits so that it is regarded as a valuable genetic resource, which deserves to be efficiently utilized for scientific conservation and commercial exploitation.

In recent years, the demand in China for pork products has been growing continuously because of the steady rise in China’s population and the rapid social development (McOrist et al., 2011). Although DSE pigs have better meat quality than Western pigs, they have the characteristic of lower growth rate that cannot meet the market demands (Wang et al., 2015). The extensive introduction of leaner Western breeds into China in the latter part of the last century resulted in a sharp decline in the number of DSE pigs. It has been more difficult to find large populations of DSE pigs. Faced with the condition mentioned above, the Chinese central government had launched a national conservation program for protecting DSE pigs (National Commission of Animal Genetic Resources of China, 2011). However, the outbreak of African swine fever disease had made the conservation get into trouble. At the same time, such severe challenges make us realize that protecting DSE pigs is an urgent and critical task.

Nowadays, it is convenient to obtain genomic information based on the high-throughput sequencing techniques; the researches on DSE pigs are not limited to phenotypic traits anymore. Exploring the genetic characterization of DSE pigs could help for preserving genomic variability, advancing scientific conservation, and contributing to sustainability (Weitzman, 1993). Population genetic data, such as single-nucleotide polymorphism (SNP), are often used to demonstrate population structure and genetic diversity in some other Chinese indigenous pigs, such as Jiangxi native pigs, Henan native pigs, Taihu native pigs, and so on (Wang et al., 2018; Qiao et al., 2019; Zhao et al., 2019). But limited genetic information–associated literatures were reported about DSE pigs, which reflected that people know little about genetic characteristics of DSE pigs. The unique phenotypic characterizations of DSE pigs were formed through long-term natural and artificial selections so that the associated selective signatures need to be detected as well, which might provide clues about the molecular mechanisms of specific traits. Both the runs of homozygosity (ROH) islands and integrated haplotype homozygosity score (iHS) methods have been identified as suitable methods for detecting selection signatures within a single breed (Chen et al., 2018; Xu et al., 2019). Therefore, with the aim to promote efficient conservation and sustainable development of DSE pigs, we describe in detail the population structure, genetic diversity, inbreeding coefficients, and selection signatures by using high-density SNPs within DSE pigs.

## Materials and Methods

### Sample Collection

A total of 75 healthy DSE pigs were selected randomly from three different regions of Yunnan Province in the present study. Among them, 27 were obtained from Yingjiang County, 29 were obtained from Jinping County, and the others were from Sipsongpanna. First, we extracted genomic DNA from ear tissues following the manufacturer’s instructions using a commercial kit (LifeFeng Biotech Co., Ltd., Shanghai, China). Subsequently, these individuals were sequenced and genotyped using the genome reducing and sequencing (GGRS) protocol. The experimental procedure for the operation of GGRS was elucidated in detail by Chen et al. (2013).

### Sequencing Data Analysis

The DNA libraries constructed with fragments ranging from 200 to 300 bp were sequenced on the Illumina Hiseq platform. The quality control of raw fastq files was performed using the NGS QC Toolkit v2.3.3 with default parameters (Patel and Jain, 2012). Then, the clean reads were mapped to the pig reference genome (Sscrofa11.1) through BWA v0.7.5, and the parameters were set according to Li and Durbin (2009). After that, SAMTOOLS v0.1.19 was used to call SNPs (Li et al., 2009), and the missing genotypes were imputed by BEAGLE v4.1 (Browning and Browning, 2016). The final SNP dataset for further analysis was obtained following the filtered criteria as follows: (1) calling quality > *Q*_20_, (2) sequencing depth > 5×, (3) minor allele frequency > 0.05, and (4) eliminate the SNP on X/Y chromosomes.

### Population Structure Analysis

To illustrate the population structure of DSE pigs, the following methods were performed: (1) the population structure based on the information from all the SNPs was performed using ADMIXTURE v1.3.0 (Alexander et al., 2009). The number of ancestral clusters (K) was set from 2 to 3, and fivefold cross-validation was run to determine the K value with the lowest cross-validation error. (2) Principal component analysis (PCA) was conducted using Plink v1.9 (command –pca 2); the first two dimensions were used to distinguish population structure. And the results of structure and PCA were visualized using R package “barplot” and “ggplot2,” respectively.

### Genetic Diversity Analysis

The effective population size (Ne), proportion of polymorphic markers (P_N_), observed heterozygosity (H_O_), expected heterozygosity (H_E_), and allelic richness (Ar) were used to investigate the genetic diversity of DSE pigs. Ne was calculated using the equation put forward by Sved (1971): *r*^2^ = 1/(4*cNe* + 1), where *c* expressed in Morgans is the genetic distance converted from the physical distance between two SNPs with the simple assumption of 1 cM ∼ 1 Mb (Uimari and Tapio, 2011); linkage disequilibrium value *r*^2^ was calculated using Plink v1.9. P_N_ was the ratio of the number of SNPs in each subgroup to the total number of SNPs. H_O_ and H_E_ were computed at the base of the SNP information through Plink v1.9. Ar was calculated using ADZE v1.0, which had the ability to correct for unequal sample size (Hurlbert, 1971; Kalinowski, 2004; Szpiech et al., 2008).

### Detection of Runs of Homozygosity

Runs of homozygosity (ROH) were estimated for individuals using a sliding window approach of 50 SNPs in Plink v1.9 with command –homozyg (Purcell et al., 2007). The parameters used to detect ROH were as follows: (i) one heterozygote was allowed in a window; (ii) two missing calls were allowed in a window; (iii) the minimum SNP density per ROH was set as 1 SNP per 50 kb; (iv) the minimum number of consecutive SNPs per ROH was set to 100; and (v) the minimum length for an ROH was set to 1 Mb. According to the physical length, we classified ROH into four different categories: > 10, 5–10, 1–5, and > 1 Mb. For each of the ROH length categories, the number of ROH per subgroup was calculated by summing all ROH per animal in that category. Besides, the frequency of ROH numbers and the total length of ROH were computed as well.

### Inbreeding Coefficient

The inbreeding coefficient based on ROH (*F*_ROH_) was calculated for individuals using the formula below (McQuillan et al., 2008):

*F*_RON_ = $\frac{L\,\text{ROH}}{L\,\text{AUTO}}$, where *L*_ROH_ is the length of autosomes covered by ROHs, and *L*_AUTO_ is the length of autosomes covered by SNPs, which was 2.26 Gb in our study. Thompson (2013) had reported that the physical length of an ROH (Mb) = 100/2 g cM, where *g* represents the number of generations of interest. Four ROH length categories were determined so that the analysis of inbreeding coefficients based on ROH length would provide information on the inbreeding during four different time spans (*F*_ROH__>__10__Mb_, *F*_ROH__5__–__10__Mb_, *F*_ROH__1__–__5__Mb_, and *F*_ROH__>__1__Mb_), corresponding to five generations ago, 5–10 generations ago, 10–50 generations ago, and 50 generations ago, respectively.

### Detection of ROH Island

To identify the genomic regions most associated with ROH, we calculated the percentage of the occurrence of SNPs in ROH by counting the number of times an SNP was detected in an ROH across individuals. The top 0.5% of SNPs showing the percentage higher than 20% were selected, and the adjacent SNPs were merged into genomic regions corresponding to ROH islands for subsequent analyses (Pemberton et al., 2012). The percentage of SNPs residing within an ROH was visualized using R package “ggplot2.” The genes within each ROH island were further extracted using R package “biomaRT” (Durinck et al., 2005).

### Selection Signature

The selection signatures in genomes within DSE pigs were detected using iHS statistic, which is powerful to identify putative regions of recent or ongoing positive selection genomes (Voight et al., 2006). The haplotype data files were derived from the phasing program fastPHASE v1.4.0 with the default parameters (Scheet and Stephens, 2006). Then, the iHS scores were computed for each autosomal SNP using the R package “rehh v3.1” (Gautier et al., 2017). The iHS statistic measures the amount of extended haplotype homozygosity for a given SNP along the ancestral allele relative to the derived allele. In this study, the ancestral alleles required for the computation of iHS were inferred as the most common alleles in the entire dataset as described by Bahbahani et al. (2015) and Bertolini et al. (2018). To calculate the *p*-value at the genomic level, iHS scores for each SNP were further transformed as *p*_iHS_ = −*log*_10_⁡(2Φ(−|iHS|)), where Φ(*x*) represents the Gaussian cumulative distribution function, and *p*_iHS_ is a two-sided *p*-value (on a −*log*_10_⁡*scale*) of a test on the null hypothesis of no selection (Gautier and Naves, 2011). Considering that the threshold was *q* = 0.05, the *p*_iHS_ scores higher than 4.24 (*q* < 0.05) were considered as putative signatures of selection.

### Functional Annotation

Common SNPs between the ROH island and iHS methods were regarded as candidate selection signatures. The Ensembl Genes 89 (Sscrofa11.1) database was used to retrieve the candidate genes that were associated with selective signatures through Ensembl Variant Effect Predictor test^1^. To further analyze the function of these candidate genes, Gene Ontology (GO) and Kyoto Encyclopedia of Gene and Genomes (KEGG) pathway enrichment analysis were performed using KOBAS 3.0^2^. The GO terms and KEGG pathways with *q* < 0.05 were regarded as significant results. Moreover, the pig quantitative trait locus (QTL) database^3^ was used to confirm the most plausible trait-associated selective signatures. The threshold length of the QTL regions was set to 1 Mb for accuracy.

## Results

The sequencing data revealed more than 380 million raw reads generated in this study, in which more than 320 million were clean reads. On average, approximately 4.35 million clean reads of each animal were detected. The average sequencing coverage and sequencing depth of the entire genome were 3.47% and 6.09×, respectively (Table 1). After quality control and filtering of unqualified SNPs, a total of 443,703 SNPs were obtained. When these SNPs were annotated to the Sscrofa11.1 genome in the Ensembl Gene database, we found that 80,307 SNPs were novel. Generally, these SNPs were distributed uniformly across the genome (except Chr Y), which can represent the information of the whole genome (Supplementary Figure S1). We discarded the SNPs on sex chromosomes, and the subsequent statistical analyses were performed using 438,038 autosomal SNPs.

**TABLE 1:** The information of sequencing data.

**Subgroups**	**No.**	**Raw reads**	**Clean reads**	**Average coverage**	**Average depth**
Yingjiang	27	164,926,919	128,935,797	3.47%	6.67 ×
Jinping	29	163,182,786	142,451,137	3.81%	6.73 ×
Sipsongpanna	19	58,244,244	54,859,021	3.14%	4.86 ×
Total	75	386,353,949	326,235,955		

**FIGURE 1:**
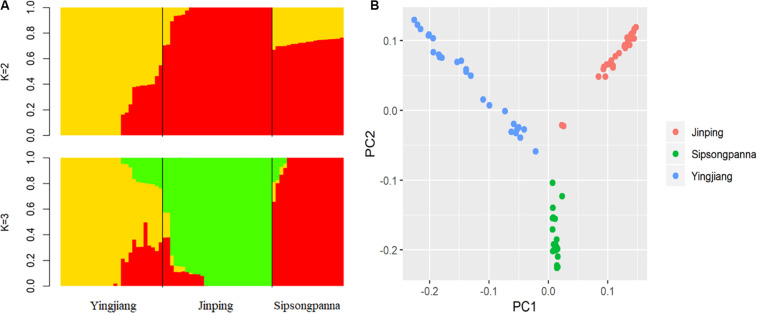
**(A)** Shows admixture plot comprising ancestry numbers (K) at 2 and 3 of all the individuals; **(B)** shows principal components analysis of all individuals, and the *x*-axis denotes the first principal component, whereas *y*-axis represents the second principal component.

An overview of the relationships among these pigs from different regions, which belong to DSE pig breed, is presented in Figure 1. When *K* = 2, the DSE pigs from Yingjiang were obviously distinguished from those from Jinping and Sipsongpanna. When *K* = 3, the DSE pigs from different regions were separated clearly (Figure 1A). The estimated *K* for the admixture analysis with the lowest cross-validation error was 2. According to the results of PCA analysis, PC1 accounted for 8.0% of the total variance, whereas PC2 accounted for 5.3% of the total variance. The first two dimensions divided these individuals into three different clades, which were identical to the geographical information of the DSE pigs (Figure 1B).

The results of genetic diversity in the three subgroups of DSE pigs are shown in Table 2. The results showed that the DSE pigs had low Ne and heterozygosity values. By comparison, we found that the Ne and P_N_ values of Yingjiang are higher than those of Jinping and Sipsongpanna, but Jinping has the highest heterozygosity ratio and Ar value among three subgroups. Overall, the value of H_E_ was always greater than the value of H_O_ in each subgroup. In addition, Sipsongpanna has the least values of each parameter of genetic diversity all the time when compared with other two subgroups, which means that the Sipsongpanna subgroup owns the lowest genetic diversity levels.

**TABLE 2:** Genetic diversity of DSE pigs.

**Subgroups**	**No.**	**Nsnp**	**Indices of genetic diversity**				
			**Ne**	**P_N_**	**H_O_**	**H_E_**	**Ar**
Yingjiang	27	433,160	46	0.988	0.219	0.289	1.873
Jinping	29	410,454	42	0.936	0.247	0.295	1.890
Sipsongpanna	19	387,882	39	0.885	0.202	0.284	1.799
All DSE pigs	75	438,308					

**No., number of individuals; Nsnp, number of SNPs; Ne, effective population size; P_N_, the proportion of polymorphic markers; H_O_, observed heterozygosity; H_E_, expected heterozygosity; Ar, allelic richness*.*

**FIGURE 2:**
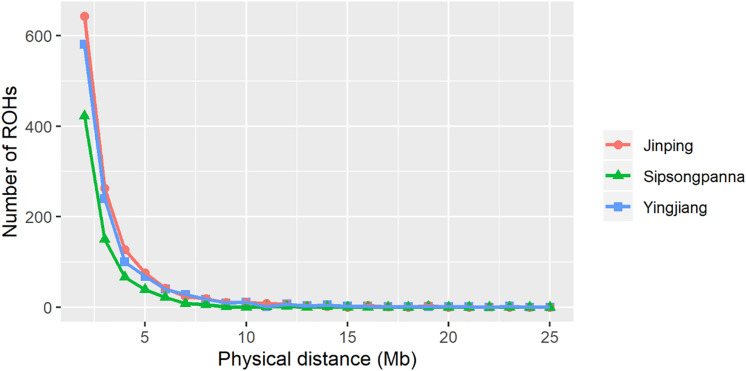
Relationship between the number of ROHs and the length of each ROH. The *x*-axis denotes the physical distance of ROHs (Mb), and the *y*-axis represents the number of ROHs.

The physical length category of ROH and the average inbreeding coefficients estimated based on the length of ROH are shown in Table 3. The distribution of ROH according to length is shown in Figure 2. A total of 1,122, 1,244, and 720 ROHs were retained from Yingjiang, Jinping, and Sipsongpanna, respectively. The length of ROH mainly fell within 1–5 Mb, and the number of ROH within 1–5 Mb accounted for more than 88% of the total number of ROH in each subgroup. In contrast, the percentages of the larger ROH (>10 Mb) were no more than 3%, especially in Sipsongpanna, which were only 0.69%. Although the percentages of larger ROH are low, they still covered a considerable portion of the total ROH length. In addition, the *F*_ROH_ based on larger segments expressed lower values than that based on smaller segments. In general, the *F*_ROH_ based on different ROH length category between Yingjiang and Jinping were always similar. Compared with others, Sipsongpanna expressed the lowest inbreeding levels.

**TABLE 3:** Inbreeding coefficients of DSE pigs.

**ROH length (Mb)**		**Yingjiang**				**Jinping**				**Sipsongpanna**		
	**No. ROH (%)**	**Total length (Mb) (%)**	**Mean length (Mb)/pig**	***F*_ROH_**	**No. ROH (%)**	**Total length (Mb) (%)**	**Mean length (Mb)/pig**	***F*_ROH_**	**No. ROH (%)**	**Total length (Mb) (%)**	**Mean length (Mb)/pig**	***F*_ROH_**
1–5	989 (88.15)	2,050.4 (64.93)	75.9	0.034	1,109 (89.15)	2,318.1 (67.66)	79.9	0.035	678 (94.17%)	1,351.6 (82.27)	71.1	0.031
5–10	106 (9.45)	715.4 (22.66)	26.5	0.012	105 (8.44)	704.3 (20.56)	24.3	0.011	37 (5.14%)	225.3 (13.71)	11.9	0.005
>10	27 (2.41)	391.9 (12.41)	14.5	0.006	30 (2.41)	403.8 (11.79)	13.9	0.006	5 (0.69%)	65.9 (4.01)	3.5	0.002
>1	1,122 (100)	3,157.7 (100)	117.0	0.052	1,244 (100)	3,426.2 (100)	118.1	0.052	720 (100%)	1,642.8 (100)	86.5	0.038

**No. ROH, number of ROH; F_*ROH*_, inbreeding coefficients based on ROH.**

**FIGURE 3:**
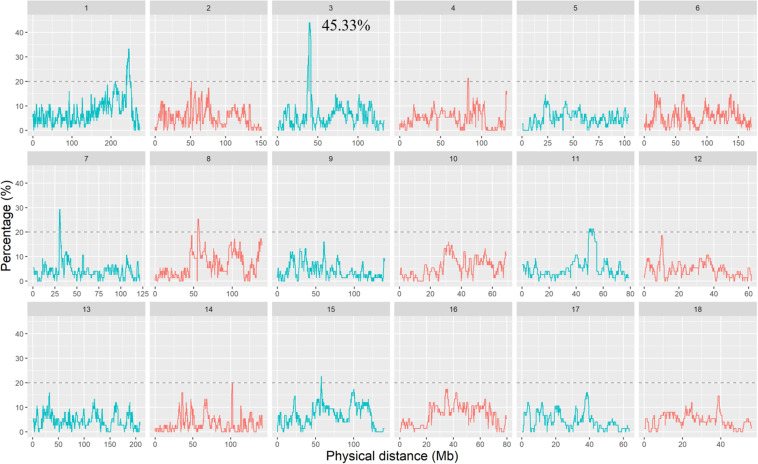
The occurrences (%) of an SNP in ROHs within DSE population. The dashed line indicates the top 0.5% threshold, which defines ROH islands.

The percentage of SNPs residing in the ROH regions across different individuals is shown in Figure 3. More than 85% of SNPs occurred with ROH of at least one individual, and the largest percentage of SNP detected in the ROH was 45.33%. In addition, we found that the frequency of different SNPs occurring within ROH regions was not uniform across the genome. To identify the ROH islands, we selected the top 0.5% of SNPs with a minimum percentage of 20%. A total of 19 ROH islands were obtained and are listed in Supplementary Table S1. The lengths of these ROH islands were distinct, ranging from 150 kb on SSC11 to 8.37 Mb on SSC1. There were more ROH islands located on SSC1 than on other autosomes. Moreover, a total of 449 genes inside these ROH islands were annotated and are provided in Supplementary Table S1. There was no correlation between the length of ROH islands and the number of genes within the ROH islands. For example, the ROH island on SSC3: 39193050–41594976 was not the longest but contained the most annotated genes. However, the ROH island on SSC11: 51567712–51718380, no gene was annotated, although it was longer than 150 kb.

**FIGURE 4:**
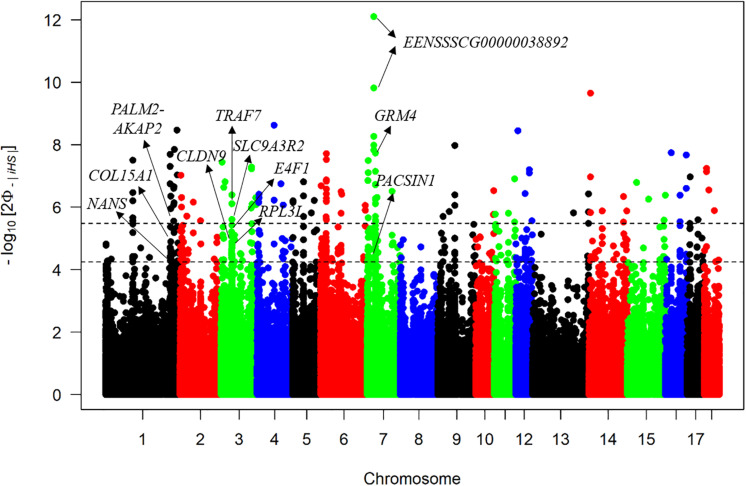
Manhattan plot of iHS test. The *x*-axis denotes the chromosome position, and the *y*-axis represents the associated *p*-value of each |iHS| score. The upper dashed line represents the threshold with *q* < 0.01, and the lower dashed line represents the threshold with *q* < 0.05.

The iHS method has strong potential to explore selective signatures within the DSE pig population. The average |iHS| value across the genome was 0.77, but the maximum |iHS| value was 7.16, which indicated that the distribution of selective signatures across the genome was non-uniform. A total of 501 significant SNPs based on |iHS| scores were detected with a *q* < 0.05, and the first two most significant outliers were both located in a novel gene ENSSSCG00000038892 on SSC7 (Figure 4). Of these, 70 significant SNPs were also detected in the ROH islands. As candidate selective signatures, Supplementary Table S2 lists the chromosome position, the percentage of SNPs in ROHs, and |iHS| value of each SNP. Finally, 20 candidate genes were identified by annotating these 70 common SNPs to the pig genome, including *NANS*, *GABBR2*, *PACSIN1*, and so on (Supplementary Table S2). Furthermore, these 20 annotated genes were used for the GO, KEGG, and QTL annotation analyses to explore related biofunctions. The most enriched pathway was “taste transduction,” which provoked our interest (Supplementary Table S3).

## Discussion

Diannan small-ear pig, one of the Chinese indigenous pigs, has excellent characteristics such as good meat quality, high intramuscular fat content, and high resistance. However, the population sizes of DSE pigs were decreased within years because of the increased introduction of Western pigs and inappropriate management. And the researches focused on the genetic information of DSE pigs were limited, which was disadvantageous to scientific conservation and commercial development for DSE pigs. Nowadays, it is difficult to obtain a large-scale population with pure DSE pigs. In our study, we randomly selected 75 DSE pigs from three different regions, and the genetic characterization of DSE pigs was analyzed using SNP information. Both the population admixture and PCA analysis implied a distinct difference among the DSE pigs from different regions. Interestingly, the distribution of DSE pigs reflected in the PCA was corresponding to their geographical origins. Such phenomenon revealed that the genetic differentiation occurred within DSE pigs in the past because of the geographic isolation. And similar discovery was also made in another study of Chinese indigenous pig breeds (Wang et al., 2018). Besides, compared with Jinping and Sipsongpanna, the distribution of Yingjiang was more scattered, which reflected the larger genetic differences among individuals in Yingjiang. According to structure analysis, *K* = 2 owned the lowest cross-validation error. When *K* = 2, Yingjiang was separated from Jinping and Sipsongpanna distinguishingly, which indicated that the level of genetic differentiation of Yingjiang was higher than that of Jinping and Sipsongpanna. Because DSE pig is a precious genetic resource, the most important for us is to strengthen conservation for DSE pigs rather than promote differentiation within DSE pigs. In consideration of these results, we need to further strengthen genetic connection among DSE pigs from different regions to avoid population genetic differentiation and promote genetic conservation for DSE pigs.

Studying genetic diversity is essential for specific population conservations, and different genetic diversity indicators display different sensitivities. Effective population size (Ne) is one of the most important concepts introduced into population genetics and plays a central part in animal breeding and conservation biology. Simon (1999) had reported that if the Ne is lower than 65, those pig breeds could be classified as being at risk. Based on the low Ne estimated in our study, the DSE pig may be in danger of extinction, and as a valuable Chinese indigenous breed, it should be included in special conservation programs for sustainable development. Heterozygosity within a population is another important index of genetic diversity. High heterozygosity means rich genetic variability, whereas low heterozygosity means poor genetic variability. Usually, we tend to attribute the discrepancy to inbreeding when H_E_ is greater than H_O_. And the heterozygosity values reported in DSE pigs are considerably lower than those reported previously for other Chinese pig breeds (Fan et al., 2002; Luetkemeier et al., 2010). Besides, the analysis of the distribution of alleles across populations is important for elucidating genetic diversity as well. Ar provides complementary information to gene diversity. The results presented in this study reflected that DSE pigs had less Ar compared with Taihu pigs (Zhao et al., 2019). In general, DSE pigs expressed the lower genetic diversity than that of some other Chinese indigenous pigs, which prompted us to be conscious of the importance of accelerating scientific conservation of DSE pigs. Therefore, we surmise whether it is more feasible to combine all the subgroups for DSE pig conservation rather than separate one breed into several subgroups. Of course, strengthening effective cooperation across different regional farms is required.

Another aspect of interest while doing research on population conservation is to study the level of inbreeding. Traditional estimation of the inbreeding coefficient is based on pedigree data. Previous studies have elucidated that using genomic data to evaluate the extent of inbreeding was more accurate than using pedigree data (Purfield et al., 2012; Zanella et al., 2016) because the pedigree data were always incomplete and failed to capture the influence of relatedness among individuals in the population. The level of ROH reflects the ancient and recent inbreeding history of a population, which has been widely applied to explore the extent of inbreeding in the population of any species (Deniskova et al., 2019; Xu et al., 2019; Bhati et al., 2020). As shown in previous report, the average ROH levels varied considerably among Chinese pigs, ranging from the lowest value of 20.6 Mb in wild boars to the largest value of 168 Mb in DSE pigs (Wang et al., 2018). In our results, the average ROH length ranged from the 86.5 Mb in Sipsongpanna to 118.1 Mb in Jinping. We found that the sum of ROH length per DSE pig is always larger than that of other Chinese pigs exactly, which indicated that DSE pigs might be influenced by inbreeding in recent or ancient years. Regardless of pigs or sheep, the numbers of shorter ROH were dominant all the time (Purfield et al., 2017; Xu et al., 2019). What’ more, the sum of ROHs in Sipsongpanna was less than that of other subgroups; the reason might be that the sample size of Sipsongpanna was only 19. Then, *F*_ROH_ based on different ROH length categories was calculated, respectively. Compared with Taihu pigs, the *F*_ROH__1__–__5__Mb_ values of DSE pigs were almost consistent with the inbreeding coefficients of small Meishan pigs and Shawutou pigs (Zhao et al., 2019). And *F*_ROH__>__5__Mb_ was lower than *F*_ROH__<__5__Mb_ in each DSE pig subgroup. These results revealed that the ancient (up to 50 generations ago) inbreeding had greater impacts than recent (within the last five generations) inbreeding on the genome within DSE pig population. The reason for this phenomenon might be that the inbreeding was inhibited effectively within DSE pig population under artificial selection in recent years.

As mentioned above, the most frequent SNP that occurred in ROH was 45.33%,and approximately only 85% of SNPs detected in an ROH of at least one individual. However, in 202 Jinhua pigs, the highest occurrence of SNP was 64.9%, and 27% of the SNPs comprised ROH in at least 20% of individuals (Xu et al., 2019). Besides, in 674 Chinese indigenous pigs, the highest percentage was 56.97% observed for an SNP within ROHs (Zhang et al., 2018). We realized that the occurrences of SNPs within the ROHs in this study were lower than the results from other researches. The reason for this difference might be due to either the fewer samples in our study or greater variation in the DSE pig population.

To improve the accuracy of the ROH islands detected in this study, we checked whether these ROH islands overlapped with the putative trait-associated genomic regions obtained in other studies. In our study, the ROH island on SSC7 partially overlapped with a selection region in a study of multiple Chinese native pig breeds, which spanned *HMGA1* and *PPARD* genes (Zhang et al., 2018). *HMGA1* and *PPARD* have been recognized as candidate genes that control limb bone length by regulating bone development (Zhang et al., 2014; Le et al., 2017). The genes *VASN*, *SEC14L5*, and *PRSS33* within different ROH islands on SSC3 (37149610–39173394, 39193050–41594976), involved in body size and lipid transport and metabolism, were also detected in the selection signature regions in Enshi black pigs (Fu et al., 2016). *PACSIN1* was also detected as a selective signature for body weight within Meishan pigs (Sun et al., 2018). In addition, there were some overlapping regions between ROH islands and QTL regions, which were associated with meat quality, growth, and immunity traits. For instance, positions 247,188,770 to 247,470,022 bp on SSC1 (ID = 8418) are a significant QTL region associated with drip loss (Ponsuksili et al., 2008). In conclusion, the ROHs across the genome in the DSE pig population are related to important economic traits under selection.

Integrated haplotype homozygosity score statistic is powerful to detect selection signatures within a single population and has been widely applied to many species (Fleming et al., 2017; Chen et al., 2018; Alshawi et al., 2019). In our study, a total of 70 SNPs were obtained through ROH islands and iHS methods. Twenty potential genes were annotated according to these SNPs, and those specific trait-associated genes that we showed were paid more attention to. For example, there was a study that showed the loss of *COL15A1* provoked muscle atrophy (Guillon et al., 2016). Another study showed that overexpression of *RPL3L* impaired the growth and myogenic fusion of myotubes (Chaillou, 2019). Jiugang et al. (2011) found that *SLC9A3R2* was differentially expressed in longissimus muscle tissues from Meishan and Large White pigs. It is well known that DSE pigs have a better taste than Western commercial pigs, which may reflect the different patterns of muscle development. Thus, we considered *COL15A1*, *RPL3L*, and *SLC9A3R2* to be the candidate genes for meat quality. In addition, *PALM2-AKAP2* was a potential locus associated with height according to the previous GWAS for the Korean population (Kim et al., 2010). Both biallelic deleterious mutations in *NANS* and *de novo* missense variants in TRAF7 have been reported to be associated with developmental delay or severe skeletal dysplasia (van Karnebeek et al., 2016; Tokita et al., 2018). *PACSIN1* was also detected as a causal gene for body weight in Meishan pigs (Sun et al., 2018). Meanwhile, the *PACSIN1* gene was also located in the body length–associated QTL region. These results suggest that *PALM2-AKAP2*, *NANS*, *TRAF7*, and *PACSIN1* might be plausible genes for body size of DSE pigs. There were also some studies that identified *CLDN9* played important roles for maintaining barrier function in airway epithelial cells and promoting lung cancer metastasis (Sharma et al., 2016; Gon et al., 2017). *E4F1* is essential for skin homeostasis because *E4F1* knockout mice suffer from skin homeostasis defects, followed by loss of cellularity in the epidermis and severe skin ulcerations (Lacroix et al., 2010). Moreover, both the selection regions associated with the *CLDN9* and *E4F1* genes were also located within the immune QTLs. As we know, the habitat of DSE pigs is in a subtropical region with high temperature and humidity. Therefore, we considered that *CLDN9* and *E4F1* might be the key factors for environmental adaptability of DSE pigs. Another detected gene was *GRM4*, which was demonstrated to be related to neuronal signal transduction and affects feed intake (Hou et al., 2018). According to the QTL analysis, *GRM4* was associated with average daily gain. Furthermore, the pathways “taste transduction” and “neuroactive ligand–receptor interaction” were the most enriched pathways in KEGG analysis. Thus, *GRM4* is regarded as a causal gene that could influence the appetite of DSE pigs.

We were aware that the sample size in our study was small for reliable estimation. Nevertheless, the sample sizes of populations in the present study were comparable to those in similar studies, which were appropriate for analyses on population structure, genetic diversity, inbreeding coefficients, and selection signatures (Ai et al., 2013; Lukić et al., 2020). Future work on a larger sample size should estimate these parameters again because they are important for conservation assessment and sustainable development of DSE pigs.

In summary, we detected that the genetic differentiation occurred within DSE pigs because of geographical isolation according to population structure and PCA analysis. Diannan small-ear pigs expressed low genetic diversity, which encouraged the breeding farms to take more intensive conservation measures for DSE pig conservation. At last, some candidate genes that may underlie differences in adaptation to specific environments and productive systems were identified in potentially selected regions. This study focused on the genetic diversity and selection signature of DSE pigs. Our findings may contribute to the strength of the conservation and sustainable development of DSE pigs and promote the understanding of the formation mechanisms of specific traits of DSE pigs.

## Data Availability Statement

Raw sequencing data that support the findings of this study have been deposited to the NCBI BioProject database under accession PRJNA639223.

## Ethics Statement

The procedures involving animals were conducted in accordance with the Chinese guidelines for animal welfare and approved by the Animal Care and Use Committee of Zhejiang University, no. ZJU20160346.

## Author Contributions

YP and QW conceived the experiments. SL, DY, XG, QC, ML, XW, and XD collected the samples. HS designed and performed the experiments. FW analyzed the data and wrote the manuscript. ZYZ, ZX, ZZ, and ZW analyzed the data. QW and QQ revised the manuscript. All authors contributed to the article and approved the submitted version.

## Conflict of Interest

The authors declare that the research was conducted in the absence of any commercial or financial relationships that could be construed as a potential conflict of interest.

## Footnotes

1 http://www.ensembl.org/info/docs/tools/vep/index.html

2 http://kobas.cbi.pku.edu.cn/anno_iden.php

3 https://www.animalgenome.org/cgi-bin/QTLdb/SS/index

## Supplementary Material

FIGURE S1 Distribution of the SNPs on the chromosomes. The *x*-axis denotes the chromosomal position (Mb), and the *y*-axis represents the chromosomes. The number of the SNPs present in each 400 kb genome block is expressed via colors.

TABLE S1 The associated genes within ROH islands.

TABLE S2 The 70 common SNPs and associated genes detected by ROH island and iHS (*q*-value< 0.05) methods.

TABLE S3 Enriched GO and KEGG pathway (*q*-value< 0.05) of candidate genes.
