# Supplementary figures and images for: Genetic Diversity and Selection Signatures Within Diannan Small-Ear Pigs Revealed by Next-Generation Sequencing
Source: Front Genet. 2020 Jul 30;11:733. doi: 10.3389/fgene.2020.00733 (PMC7406676; doi:10.3389/fgene.2020.00733)

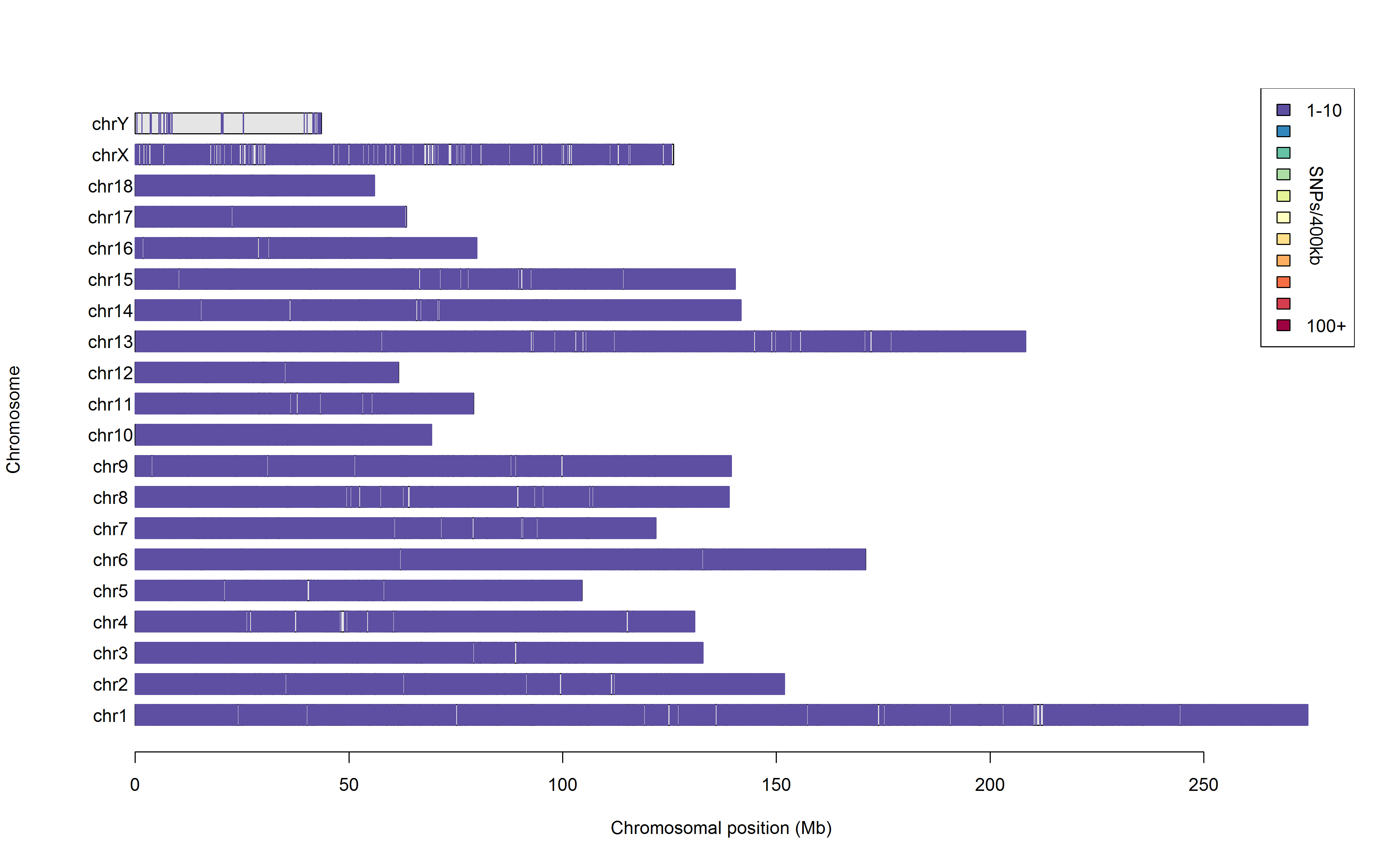

Supplement: FIGURE S1 — Distribution of the SNPs on the chromosomes. The x-axis denotes the chromosomal position (Mb), and the y-axis represents the chromosomes. The number of the SNPs present in each 400 kb genome block is expressed via colors. [file Image_1.TIF]
