# Supplementary material for: Genetic Diversity and Selection Signatures Within Diannan Small-Ear Pigs Revealed by Next-Generation Sequencing
Source: Front Genet. 2020 Jul 30;11:733. doi: 10.3389/fgene.2020.00733 (PMC7406676; doi:10.3389/fgene.2020.00733)
Supplement: TABLE S1 — The associated genes within ROH islands. [file Data_Sheet_1.docx]

Table S1 The associated genes within ROH islands

| SSC | Start position | End position | Width (bp) | No. of Gene | Gene ID | Gene Symbol |
| --- | --- | --- | --- | --- | --- | --- |
| 1 | 210,832,883 | 211,686,104 | 853,222 | 3 | ENSSSCG00000047464  ENSSSCG00000050395  ENSSSCG00000050028 | -  -  - |
| 1 | 239,425,664 | 240,783,081 | 1,357,418 | 16 | ENSSSCG00000005366  ENSSSCG00000005367  ENSSSCG00000005368  ENSSSCG00000050253  ENSSSCG00000049997  ENSSSCG00000005369  ENSSSCG00000005371  ENSSSCG00000005373  ENSSSCG00000033222  ENSSSCG00000005375  ENSSSCG00000005376  ENSSSCG00000027558  ENSSSCG00000042311  ENSSSCG00000005378  ENSSSCG00000005379  ENSSSCG00000005380 | NCBP1  TSTD2  XPA  -  -  FOXE1  -  NANS  TRIM14  CORO2A  TBC1D2  GABBR2  -  ANKS6  GALNT12  COL15A1 |
| 1 | 240,801,359 | 249,178,354 | 8,376,996 | 94 | ENSSSCG00000005380  ENSSSCG00000005382  ENSSSCG00000042369  ENSSSCG00000005383  ENSSSCG00000038914  ENSSSCG00000041602  ENSSSCG00000041432  ENSSSCG00000005385  ENSSSCG00000050211  ENSSSCG00000048140  ENSSSCG00000005386  ENSSSCG00000040634  ENSSSCG00000005388  ENSSSCG00000005389  ENSSSCG00000005391  ENSSSCG00000037954  ENSSSCG00000047956  ENSSSCG00000041513  ENSSSCG00000044225  ENSSSCG00000046621  ENSSSCG00000050727  ENSSSCG00000005393  ENSSSCG00000045284  ENSSSCG00000042524  ENSSSCG00000038171  ENSSSCG00000005398  ENSSSCG00000035245  ENSSSCG00000027974  ENSSSCG00000034239  ENSSSCG00000038300  ENSSSCG00000029687  ENSSSCG00000042952  ENSSSCG00000049199  ENSSSCG00000005399  ENSSSCG00000005400  ENSSSCG00000044263  ENSSSCG00000005401  ENSSSCG00000051755  ENSSSCG00000005403  ENSSSCG00000049425  ENSSSCG00000045877  ENSSSCG00000044671  ENSSSCG00000050700  ENSSSCG00000026768  ENSSSCG00000026865  ENSSSCG00000031355  ENSSSCG00000005413  ENSSSCG00000050029  ENSSSCG00000047454  ENSSSCG00000044110  ENSSSCG00000051550  ENSSSCG00000035714  ENSSSCG00000036043  ENSSSCG00000005419  ENSSSCG00000005420  ENSSSCG00000035397  ENSSSCG00000005424  ENSSSCG00000005423  ENSSSCG00000045023  ENSSSCG00000005425  ENSSSCG00000051119  ENSSSCG00000005426  ENSSSCG00000037867  ENSSSCG00000047839  ENSSSCG00000039896  ENSSSCG00000020046  ENSSSCG00000042963  ENSSSCG00000043278  ENSSSCG00000051531  ENSSSCG00000005430  ENSSSCG00000051198  ENSSSCG00000048390  ENSSSCG00000049191  ENSSSCG00000046369  ENSSSCG00000019308  ENSSSCG00000005432  ENSSSCG00000049118  ENSSSCG00000051396  ENSSSCG00000031592  ENSSSCG00000049088  ENSSSCG00000045588  ENSSSCG00000047702  ENSSSCG00000051325  ENSSSCG00000041700  ENSSSCG00000044363  ENSSSCG00000043047  ENSSSCG00000005436  ENSSSCG00000005437  ENSSSCG00000043094  ENSSSCG00000042821  ENSSSCG00000048964  ENSSSCG00000043016  ENSSSCG00000048610  ENSSSCG00000051392 | COL15A1  TGFBR1  -  ALG2  -  -  -  NR4A3  -  -  STX17  -  INVS  TEX10  MSANTD3-TMEFF1  CAVIN4  -  -  -  -  -  PLPPR1  -  -  -  -  BAAT  MRPL50  ZNF189  ALDOB  TMEM246  -  -  RNF20  GRIN3A  -  -  -  SMC2  -  -  -  -  -  -  OR13C8  -  -  -  -  -  -  -  -  -  -  NIPSNAP3A  -  -  SLC44A1  -  FSD1L  TAL2  -  -  -  -  -  -  -  -  -  -  -  -  -  -  -  ZNF462  -  -  -  -  -  -  -  RAD23B  KLF4  -  -  -  -  -  - |
| 1 | 249259139 | 249915254 | 656116 | 7 | ENSSSCG00000041167  ENSSSCG00000031596  ENSSSCG00000050101  ENSSSCG00000005438  ENSSSCG00000005439  ENSSSCG00000005440  ENSSSCG00000005441 | -  -  -  ACTL7B  ACTL7A  -  ELP1 |
| 1 | 250007695 | 250161716 | 154022 | 4 | ENSSSCG00000005444  ENSSSCG00000018471  ENSSSCG00000028093  ENSSSCG00000048786 | TMEM245  -  -  - |
| 1 | 250638431 | 250812871 | 174441 | 1 | ENSSSCG00000033120 | PALM2-AKAP2 |
| 1 | 250826561 | 251157976 | 331416 | 1 | ENSSSCG00000033120 | PALM2-AKAP2 |
| 1 | 251165875 | 251614348 | 448474 | 8 | ENSSSCG00000033120  ENSSSCG00000005452  ENSSSCG00000036297  ENSSSCG00000005453  ENSSSCG00000045607  ENSSSCG00000005454  ENSSSCG00000005455  ENSSSCG00000038352 | PALM2-AKAP2  C9orf152  -  -  -  -  SVEP1  - |
| 2 | 51245196 | 51455995 | 210800 | 5 | ENSSSCG00000029050  ENSSSCG00000013990  ENSSSCG00000013989  ENSSSCG00000013988  ENSSSCG00000034569 | MRPL55  GUK1  GJC2  -  - |
| 3 | 37149610 | 39173394 | 2023785 | 66 | ENSSSCG00000007917  ENSSSCG00000019447  ENSSSCG00000046716  ENSSSCG00000007920  ENSSSCG00000007919  ENSSSCG00000025072  ENSSSCG00000039066  ENSSSCG00000007925  ENSSSCG00000007926  ENSSSCG00000007927  ENSSSCG00000007924  ENSSSCG00000007931  ENSSSCG00000007932  ENSSSCG00000007933  ENSSSCG00000007939  ENSSSCG00000048144  ENSSSCG00000007935  ENSSSCG00000035999  ENSSSCG00000007930  ENSSSCG00000023603  ENSSSCG00000007940  ENSSSCG00000007941  ENSSSCG00000007943  ENSSSCG00000007942  ENSSSCG00000022874  ENSSSCG00000007944  ENSSSCG00000025561  ENSSSCG00000007947  ENSSSCG00000023653  ENSSSCG00000046837  ENSSSCG00000050892  ENSSSCG00000040215  ENSSSCG00000007949  ENSSSCG00000007950  ENSSSCG00000018870  ENSSSCG00000007951  ENSSSCG00000007952  ENSSSCG00000036527  ENSSSCG00000007954  ENSSSCG00000007956  ENSSSCG00000007955  ENSSSCG00000021065  ENSSSCG00000007957  ENSSSCG00000033390  ENSSSCG00000007958  ENSSSCG00000029285  ENSSSCG00000037309  ENSSSCG00000007963  ENSSSCG00000045863  ENSSSCG00000043625  ENSSSCG00000043838  ENSSSCG00000007965  ENSSSCG00000007961  ENSSSCG00000034150  ENSSSCG00000047523  ENSSSCG00000038889  ENSSSCG00000007967  ENSSSCG00000029375  ENSSSCG00000029641  ENSSSCG00000021569  ENSSSCG00000020895  ENSSSCG00000023315  ENSSSCG00000027252  ENSSSCG00000027130  ENSSSCG00000023743  ENSSSCG00000021536 | RBFOX1  -  -  -  -  C16orf89  -  NAGPA  SEC14L5  PPL  UBN1  GLYR1  ROGDI  SEPTIN12  ANKS3  -  -  NUDT16L1  MGRN1  UBALD1  -  CDIP1  NMRAL1  HMOX2  DNAJA3  -  VASN  PAM16  GLIS2  -  -  TFAP4  SRL  ADCY9  -  CREBBP  -  DNASE1  SLX4  NLRC3  CLUAP1  NAA60  C16orf90  -  ZNF174  -  TIGD7  ZNF263  -  -  -  ZNF200  -  -  -  -  ZNF213  ZNF205  ZSCAN10  MMP25  BICDL2  THOC6  HCFC1R1  -  CLDN6  CLDN9 |
| 3 | 39193050 | 41594976 | 2401927 | 145 | ENSSSCG00000026248  ENSSSCG00000029264  ENSSSCG00000035052  ENSSSCG00000028381  ENSSSCG00000022630  ENSSSCG00000036096  ENSSSCG00000023304  ENSSSCG00000045106  ENSSSCG00000008061  ENSSSCG00000030518  ENSSSCG00000008063  ENSSSCG00000022744  ENSSSCG00000033610  ENSSSCG00000037112  ENSSSCG00000035594  ENSSSCG00000008062  ENSSSCG00000008058  ENSSSCG00000037719  ENSSSCG00000008057  ENSSSCG00000008056  ENSSSCG00000027098  ENSSSCG00000008054  ENSSSCG00000008055  ENSSSCG00000026326  ENSSSCG00000031982  ENSSSCG00000008052  ENSSSCG00000008051  ENSSSCG00000008048  ENSSSCG00000008047  ENSSSCG00000024587  ENSSSCG00000036614  ENSSSCG00000008044  ENSSSCG00000042435  ENSSSCG00000008045  ENSSSCG00000033059  ENSSSCG00000008043  ENSSSCG00000031232  ENSSSCG00000033691  ENSSSCG00000047720  ENSSSCG00000008041  ENSSSCG00000008040  ENSSSCG00000008039  ENSSSCG00000008038  ENSSSCG00000048627  ENSSSCG00000023400  ENSSSCG00000008036  ENSSSCG00000008035  ENSSSCG00000008034  ENSSSCG00000037686  ENSSSCG00000033697  ENSSSCG00000034728  ENSSSCG00000036739  ENSSSCG00000026828  ENSSSCG00000027755  ENSSSCG00000030328  ENSSSCG00000032657  ENSSSCG00000031216  ENSSSCG00000032932  ENSSSCG00000045678  ENSSSCG00000025711  ENSSSCG00000008012  ENSSSCG00000039715  ENSSSCG00000008013  ENSSSCG00000008014  ENSSSCG00000039024  ENSSSCG00000021467  ENSSSCG00000008016  ENSSSCG00000008017  ENSSSCG00000008018  ENSSSCG00000008019  ENSSSCG00000008026  ENSSSCG00000008020  ENSSSCG00000008021  ENSSSCG00000008022  ENSSSCG00000037262  ENSSSCG00000043968  ENSSSCG00000008024  ENSSSCG00000028139  ENSSSCG00000048138  ENSSSCG00000038882  ENSSSCG00000022963  ENSSSCG00000008031  ENSSSCG00000008029  ENSSSCG00000008030  ENSSSCG00000021560  ENSSSCG00000050063  ENSSSCG00000008032  ENSSSCG00000021222  ENSSSCG00000035337  ENSSSCG00000034266  ENSSSCG00000037970  ENSSSCG00000032076  ENSSSCG00000032400  ENSSSCG00000033234  ENSSSCG00000008009  ENSSSCG00000041715  ENSSSCG00000031625  ENSSSCG00000050016  ENSSSCG00000047989  ENSSSCG00000008010  ENSSSCG00000008011  ENSSSCG00000008000  ENSSSCG00000023487  ENSSSCG00000008001  ENSSSCG00000008002  ENSSSCG00000008003  ENSSSCG00000050615  ENSSSCG00000008004  ENSSSCG00000008005  ENSSSCG00000008006  ENSSSCG00000008007  ENSSSCG00000007988  ENSSSCG00000008008  ENSSSCG00000032501  ENSSSCG00000007998  ENSSSCG00000007991  ENSSSCG00000007995  ENSSSCG00000032188  ENSSSCG00000037013  ENSSSCG00000033792  ENSSSCG00000007992  ENSSSCG00000007989  ENSSSCG00000007993  ENSSSCG00000035854  ENSSSCG00000044588  ENSSSCG00000007987  ENSSSCG00000007986  ENSSSCG00000007985  ENSSSCG00000007984  ENSSSCG00000023204  ENSSSCG00000007983  ENSSSCG00000038979  ENSSSCG00000036541  ENSSSCG00000007980  ENSSSCG00000007979  ENSSSCG00000038804  ENSSSCG00000007981  ENSSSCG00000007978  ENSSSCG00000007977  ENSSSCG00000007975  ENSSSCG00000007982  ENSSSCG00000007968  ENSSSCG00000007970  ENSSSCG00000007962  ENSSSCG00000007973 | -  PKMYT1  KREMEN2  -  FLYWCH2  -  SRRM2  -  ELOB  PRSS33  PRSS41  PRSS21  -  -  PRSS22  PRSS27  KCTD5  PDPK1  -  -  -  NTN3  TEDC2  CCNF  -  -  ABCA3  RNPS1  E4F1  -  PGP  MLST8  -  BRICD5  CASKIN1  TRAF7  SNORD60  RAB26  -  PKD1  TSC2  NTHL1  SLC9A3R2  -  ZNF598  SYNGR3  -  NOXO1  TBL3  RPS2  RNF151  SNORA78  SNORA64  SNORA10  NDUFB10  RPL3L  HS3ST6  -  -  MEIOB  -  FAHD1  IGFALS  NUBP2  SPSB3  EME2  -  NME3  MAPK8IP3  -  CRAMP1  IFT140  TMEM204  TELO2  PTX4  -  CLCN7  CCDC154  -  C16orf91  -  GNPTG  BAIAP3  TSR3  -  -  -  -  -  CACNA1H  SOX8  -  C1QTNF8  -  LMF1  -  -  -  -  CHTF18  GNG13  RPUSD1  MSLNL  -  CIAO3  HAGHL  CCDC78  ANTKMT  -  FBXL16  WDR24  JMJD8  STUB1  -  RHBDL1  WDR90  MCRIP2  METTL26  WFIKKN1  RAB40C  PIGQ  PRR35  CAPN15  RAB11FIP3  -  DECR2  NME4  TMEM8A  MRPL28  AXIN1  PDIA2  ARHGDIG  RGS11  FAM234A  LUC7L  HBQ1  NPRL3  -  HBM  HBZ  -  RHBDF1  POLR3K  SNRNP25  - |
| 4 | 83030583 | 84198911 | 1168329 | 18 | ENSSSCG00000006303  ENSSSCG00000006304  ENSSSCG00000037094  ENSSSCG00000006305  ENSSSCG00000019847  ENSSSCG00000006306  ENSSSCG00000006307  ENSSSCG00000006308  ENSSSCG00000006309  ENSSSCG00000006310  ENSSSCG00000006311  ENSSSCG00000006312  ENSSSCG00000006316  ENSSSCG00000035124  ENSSSCG00000038511  ENSSSCG00000042247  ENSSSCG00000006318  ENSSSCG00000006319 | DCAF6  MPC2  -  ADCY10  -  MPZL1  RCSD1  CREG1  CD247  POU2F1  DUSP27  GPA33  -  ILDR2  -  -  TADA1  POGK |
| 7 | 30076980 | 31375384 | 1298405 | 23 | ENSSSCG00000001523  ENSSSCG00000031593  ENSSSCG00000032242  ENSSSCG00000023160  ENSSSCG00000027053  ENSSSCG00000036573  ENSSSCG00000001527  ENSSSCG00000037476  ENSSSCG00000001531  ENSSSCG00000001532  ENSSSCG00000001533  ENSSSCG00000001534  ENSSSCG00000001535  ENSSSCG00000034982  ENSSSCG00000001536  ENSSSCG00000001537  ENSSSCG00000001538  ENSSSCG00000001539  ENSSSCG00000042446  ENSSSCG00000001546  ENSSSCG00000001543  ENSSSCG00000001544  ENSSSCG00000035972 | GRM4  -  HMGA1  -  PACSIN1  SPDEF  -  -  SNRPC  UHRF1BP1  TAF11  ANKS1A  TCP11  -  SCUBE3  ZNF76  DEF6  PPARD  -  FANCE  -  -  TULP1 |
| 8 | 55114296 | 57199599 | 2085304 | 26 | ENSSSCG00000020369  ENSSSCG00000022882  ENSSSCG00000023269  ENSSSCG00000027301  ENSSSCG00000022283  ENSSSCG00000042852  ENSSSCG00000008903  ENSSSCG00000027403  ENSSSCG00000008902  ENSSSCG00000008901  ENSSSCG00000043888  ENSSSCG00000008900  ENSSSCG00000008899  ENSSSCG00000032353  ENSSSCG00000044724  ENSSSCG00000008898  ENSSSCG00000041079  ENSSSCG00000038107  ENSSSCG00000023357  ENSSSCG00000025792  ENSSSCG00000036736  ENSSSCG00000022955  ENSSSCG00000008913  ENSSSCG00000050886  ENSSSCG00000050885  ENSSSCG00000044641 | -  EXOC1L  EXOC1  -  CEP135  -  KIAA1211  AASDH  PPAT  PAICS  -  -  ARL9  THEGL  -  -  -  -  REST  NOA1  -  POLR2B  -  -  -  - |
| 11 | 49445372 | 51451169 | 2005798 | 14 | ENSSSCG00000047458  ENSSSCG00000009474  ENSSSCG00000009475  ENSSSCG00000009477  ENSSSCG00000041521  ENSSSCG00000043223  ENSSSCG00000049459  ENSSSCG00000040027  ENSSSCG00000019130  ENSSSCG00000025334  ENSSSCG00000029113  ENSSSCG00000035748  ENSSSCG00000009479  ENSSSCG00000009480 | -  SCEL  SLAIN1  EDNRB  -  -  -  -  -  POU4F1  OBI1  -  RBM26  NDFIP2 |
| 11 | 51567712 | 51718380 | 150669 | - | - | - |
| 11 | 51922886 | 53416731 | 1493846 | 6 | ENSSSCG00000051397  ENSSSCG00000009482  ENSSSCG00000050522  ENSSSCG00000041420  ENSSSCG00000021526  ENSSSCG00000045873 | -  SPRY2  -  -  -  - |
| 14 | 101908777 | 102452061 | 543285 | 4 | ENSSSCG00000045077  ENSSSCG00000048171  ENSSSCG00000050653  ENSSSCG00000010459 | -  -  -  HTR7 |
| 15 | 57212657 | 57713785 | 501129 | 8 | ENSSSCG00000035828  ENSSSCG00000033126  ENSSSCG00000028679  ENSSSCG00000048830  ENSSSCG00000050269  ENSSSCG00000041936  ENSSSCG00000047017  ENSSSCG00000047693 | ARHGEF4  FAM168B  PLEKHB2  -  -  -  -  - |

Table S2 The 70 common SNPs and associated genes detected by ROH island and iHS (*q*-value< 0. 05) methods

| SSC | Position | Percentage of SNP in ROH (%) | \|iHS\| value | *q*-value | Gene ID | Gene Symbol | QTL trait |
| --- | --- | --- | --- | --- | --- | --- | --- |
| 1 | 239910676 | 22.67 | 4.055856 | 0.047 | ENSSSCG00000005373 | NANS |  |
| 1 | 240130317 | 22.67 | 4.066535 | 0.046 | ENSSSCG00000027558 | GABBR2 |  |
| 1 | 240180878 | 26.67 | 4.169994 | 0.037 | ENSSSCG00000027558 | GABBR2 |  |
| 1 | 240287067 | 26.67 | 4.231464 | 0.032 | ENSSSCG00000027558 | GABBR2 |  |
| 1 | 240652705 | 21.33 | 5.050159 | 0.003 | - | - |  |
| 1 | 240652716 | 21.33 | 4.870123 | 0.005 | - | - |  |
| 1 | 240734847 | 20.00 | 4.366377 | 0.023 | ENSSSCG00000005380 | COL15A1 | Drip loss |
| 1 | 240776550 | 21.33 | 4.040606 | 0.048 | ENSSSCG00000005380 | COL15A1 | Drip loss |
| 1 | 240812214 | 20.00 | 4.206093 | 0.034 | ENSSSCG00000005380 | COL15A1 | Drip loss |
| 1 | 240954582 | 21.33 | 4.101149 | 0.042 | - | - |  |
| 1 | 251165956 | 20.00 | 4.687953 | 0.009 | ENSSSCG00000033120 | PALM2-AKAP2 |  |
| 3 | 38800275 | 30.67 | 4.090578 | 0.044 | - | - |  |
| 3 | 38804352 | 30.67 | 4.141678 | 0.039 | - | - |  |
| 3 | 39173126 | 30.67 | 4.228129 | 0.032 | ENSSSCG00000021536 | CLDN9 | CD8-positive leukocyte percentage |
| 3 | 39197923 | 40.00 | 4.174982 | 0.036 | ENSSSCG00000026248 | - |  |
| 3 | 39760587 | 45.33 | 4.626753 | 0.011 | ENSSSCG00000008047/  ENSSSCG00000024587 | E4F1/  - | CD8-negative leukocyte percentage |
| 3 | 39781717 | 45.33 | 4.124371 | 0.041 | ENSSSCG00000008044/  ENSSSCG00000008045 | MLST8/  BRICD5 |  |
| 3 | 39793297 | 45.33 | 4.282393 | 0.028 | - | - |  |
| 3 | 39802134 | 45.33 | 4.293722 | 0.028 | ENSSSCG00000033059 | CASKIN1 |  |
| 3 | 39811682 | 45.33 | 4.106636 | 0.042 | ENSSSCG00000008043 | TRAF7 |  |
| 3 | 39815111 | 45.33 | 4.469003 | 0.017 | ENSSSCG00000008043 | TRAF7 |  |
| 3 | 39815581 | 45.33 | 5.0678 | 0.003 | ENSSSCG00000008043 | TRAF7 |  |
| 3 | 39821701 | 45.33 | 4.258362 | 0.030 | ENSSSCG00000008043 | TRAF7 |  |
| 3 | 39828405 | 45.33 | 4.941834 | 0.003 | - | - |  |
| 3 | 39828426 | 45.33 | 4.941834 | 0.003 | - | - |  |
| 3 | 39828775 | 45.33 | 4.257261 | 0.030 | - | - |  |
| 3 | 39831008 | 45.33 | 4.262873 | 0.029 | - | - |  |
| 3 | 39947736 | 45.33 | 4.44647 | 0.018 | ENSSSCG00000008038 | SLC9A3R2 |  |
| 3 | 39950721 | 45.33 | 4.709913 | 0.009 | ENSSSCG00000008038 | SLC9A3R2 |  |
| 3 | 39954446 | 45.33 | 4.356871 | 0.023 | - | - |  |
| 3 | 39975936 | 45.33 | 4.468839 | 0.017 | ENSSSCG00000008036 | SYNGR3 |  |
| 3 | 40001820 | 45.33 | 4.328556 | 0.025 | ENSSSCG00000033697 | RPS2 |  |
| 3 | 40014342 | 45.33 | 4.152033 | 0.038 | ENSSSCG00000032657 | RPL3L |  |
| 7 | 30116205 | 22.67 | 4.124966 | 0.041 | - | - |  |
| 7 | 30120110 | 22.67 | 4.617695 | 0.011 | - | - |  |
| 7 | 30120130 | 22.67 | 4.558604 | 0.013 | - | - |  |
| 7 | 30170293 | 22.67 | 4.942122 | 0.003 | - | - |  |
| 7 | 30170370 | 22.67 | 4.229698 | 0.032 | - | - |  |
| 7 | 30189382 | 24.00 | 4.27778 | 0.028 | ENSSSCG00000001523 | GRM4 | Average daily gain |
| 7 | 30194467 | 24.00 | 4.086579 | 0.044 | ENSSSCG00000001523 | GRM4 | Average daily gain |
| 7 | 30194480 | 24.00 | 4.086579 | 0.044 | ENSSSCG00000001523 | GRM4 | Average daily gain |
| 7 | 30196178 | 24.00 | 4.474768 | 0.017 | ENSSSCG00000001523 | GRM4 | Average daily gain |
| 7 | 30196202 | 24.00 | 4.753411 | 0.007 | ENSSSCG00000001523 | GRM4 | Average daily gain |
| 7 | 30202660 | 26.67 | 5.21352 | 0.002 | ENSSSCG00000001523 | GRM4 | Average daily gain |
| 7 | 30202683 | 26.67 | 5.21352 | 0.002 | ENSSSCG00000001523 | GRM4 | Average daily gain |
| 7 | 30202685 | 26.67 | 5.207294 | 0.002 | ENSSSCG00000001523 | GRM4 | Average daily gain |
| 7 | 30202726 | 26.67 | 4.999596 | 0.003 | ENSSSCG00000001523 | GRM4 | Average daily gain |
| 7 | 30204758 | 26.67 | 4.677961 | 0.009 | ENSSSCG00000001523 | GRM4 | Average daily gain |
| 7 | 30212291 | 26.67 | 4.336578 | 0.025 | ENSSSCG00000001523 | GRM4 | Average daily gain |
| 7 | 30229577 | 26.67 | 5.211916 | 0.002 | ENSSSCG00000001523 | GRM4 | Average daily gain |
| 7 | 30231858 | 26.67 | 5.017163 | 0.003 | ENSSSCG00000001523 | GRM4 | Average daily gain |
| 7 | 30232049 | 26.67 | 5.625049 | 0.001 | ENSSSCG00000001523 | GRM4 | Average daily gain |
| 7 | 30233509 | 26.67 | 4.694148 | 0.009 | ENSSSCG00000001523 | GRM4 | Average daily gain |
| 7 | 30233563 | 26.67 | 4.694148 | 0.009 | ENSSSCG00000001523 | GRM4 | Average daily gain |
| 7 | 30236179 | 26.67 | 4.699158 | 0.009 | ENSSSCG00000001523 | GRM4 | Average daily gain |
| 7 | 30236202 | 26.67 | 4.430492 | 0.019 | ENSSSCG00000001523 | GRM4 | Average daily gain |
| 7 | 30236319 | 26.67 | 4.670609 | 0.009 | ENSSSCG00000001523 | GRM4 | Average daily gain |
| 7 | 30241788 | 26.67 | 4.243975 | 0.031 | ENSSSCG00000001523 | GRM4 | Average daily gain |
| 7 | 30244432 | 26.67 | 4.45461 | 0.018 | ENSSSCG00000001523 | GRM4 | Average daily gain |
| 7 | 30488384 | 29.33 | 4.492087 | 0.016 | - | - |  |
| 7 | 30488407 | 29.33 | 4.492087 | 0.016 | - | - |  |
| 7 | 30488526 | 29.33 | 4.408419 | 0.020 | - | - |  |
| 7 | 30490290 | 29.33 | 5.387382 | 0.001 | - | - |  |
| 7 | 30490327 | 29.33 | 5.387382 | 0.001 | - | - |  |
| 7 | 30490385 | 29.33 | 5.387382 | 0.001 | - | - |  |
| 7 | 30496698 | 29.33 | 4.067793 | 0.046 | ENSSSCG00000027053 | PACSIN1 | Body length |
| 7 | 30497692 | 29.33 | 4.03995 | 0.048 | ENSSSCG00000027053 | PACSIN1 | Body length |
| 7 | 30502226 | 29.33 | 4.135828 | 0.040 | ENSSSCG00000027053 | PACSIN1 | Body length |
| 7 | 31327841 | 20.00 | 4.386288 | 0.021 | ENSSSCG00000001543/  ENSSSCG00000001546 | -/  FANCE |  |
| 14 | 102329023 | 20.00 | 4.16195 | 0.037 | - | - |  |

Table S3 Enriched GO and KEGG pathway (*q*-value < 0.05) of candidate genes

| ID | Terms | *q*-value | Genes |
| --- | --- | --- | --- |
| GO:1901564 | organonitrogen compound metabolic process | 0.010 | E4F1, GRM4, RPL3L, MLST8, TRAF7 |
| GO:0005924 | cell-substrate adherens junction | 0.010 | SLC9A3R2, RPS2 |
| GO:0003824 | catalytic activity | 0.010 | GRM4, GABBR2, RPS2, TRAF7 |
| GO:0016740 | transferase activity | 0.010 | GRM4, RPS2, TRAF7 |
| GO:0099537 | trans-synaptic signaling | 0.012 | GRM4, GABBR2 |
| GO:0034470 | ncRNA processing | 0.023 | RPL3L, RPS2 |
| GO:0099528 | G protein-coupled neurotransmitter receptor activity | 0.043 | GABBR2 |
| GO:0005887 | integral component of plasma membrane | 0.043 | GRM4, GABBR2 |
| GO:0044238 | primary metabolic process | 0.044 | GRM4, NANS, MLST8, RPS2 |
| GO:0005201 | extracellular matrix structural constituent | 0.044 | COL15A1 |
| GO:0099501 | exocytic vesicle membrane | 0.045 | SYNGR3 |
| GO:0005581 | collagen trimer | 0.049 | COL15A1 |
| GO:0022626 | cytosolic ribosome | 0.050 | RPL3L |
| GO:0043296 | apical junction complex | 0.050 | CLDN9 |
| hsa04742 | Taste transduction | 0.010 | GRM4, GABBR2 |
| hsa03010 | Ribosome | 0.022 | RPL3L, RPS2 |
| hsa04080 | Neuroactive ligand-receptor interaction | 0.049 | GRM4, GABBR2 |
